# Supplementary material for: Insights into the Adolescent Cystic Fibrosis Airway Microbiome Using Shotgun Metagenomics
Source: Int J Mol Sci. 2024 Mar 31;25(7):3893. doi: 10.3390/ijms25073893 (PMC11011389; doi:10.3390/ijms25073893)
Supplement: Supplementary file 1 [file ijms-25-03893-s001.zip › Supplementary files ZIP/Figure S1.pdf]

A

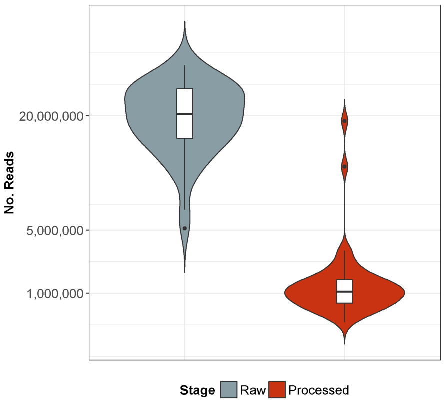

B

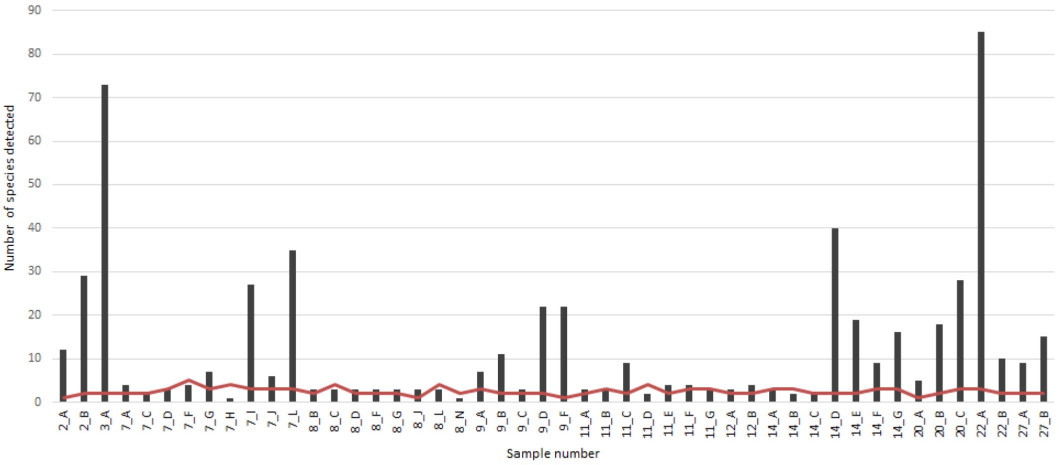

Figure S1: Sequencing metrics and quality control. A) number of raw reads versus processed reads and B) Number of species detected by NextSeq and culture. Black bars represent number of species detected by NextSeq and red line represents the number of species detected by culture for each sample.
