# Supplementary material for: Insights into the Adolescent Cystic Fibrosis Airway Microbiome Using Shotgun Metagenomics
Source: Int J Mol Sci. 2024 Mar 31;25(7):3893. doi: 10.3390/ijms25073893 (PMC11011389; doi:10.3390/ijms25073893)
Supplement: Supplementary file 1 [file ijms-25-03893-s001.zip › Supplementary files ZIP/Figure S2.pdf]

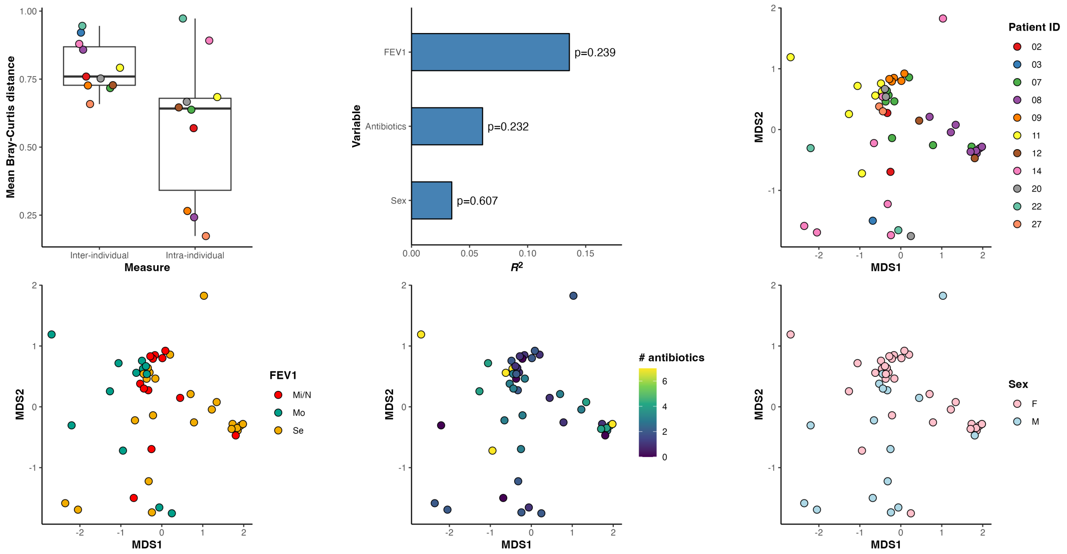

Figure S2: Beta-diversity analysis. (A) Bray-Curtis distances between samples. (B) PERMANOVA results. MDS plots coloured by (C) patient ID, (D) FEV1, (E) number of antibiotics, and (F) sex.
