# Supplementary material for: Insights into the Adolescent Cystic Fibrosis Airway Microbiome Using Shotgun Metagenomics
Source: Int J Mol Sci. 2024 Mar 31;25(7):3893. doi: 10.3390/ijms25073893 (PMC11011389; doi:10.3390/ijms25073893)
Supplement: Supplementary file 1 [file ijms-25-03893-s001.zip › Supplementary files ZIP/Table S1.pdf]

Table S1: Agars used for approximate identification of listed bacteria

Bacteria	Selective Agar	Oxygenation	Time (h)	Temp
P. aeruginosa	Cetrimide (Fannin)	Aerobic	24	37°C
S. aureus	CHROMID® *S. aureus* Elite agar (SAIDE) and *S. aureus* agar (SAID) – (Biomerieux)	Aerobic	24	37°C
H. influenzae	Chocolate agar with Bacitracin (Fannin)	Anaerobic	24	37°C
S. maltophilia	Vancomycin, Imipenem & Amphotericin B Agar (VIA agar)	Aerobic	48	37°C
A. baumannii	CHROMagar™ Acinetobacter	Aerobic	48	37°C
E. coli, Enterococcus, Klebsiella, Enterobacter, Citrobacter, Proteus, Pseudomonas, S. aureus, S. saprophyticus	CHROMagar™ Orientation	Aerobic	24	37°C
